# Gotcha GPT: Ensuring the Integrity in Academic Writing

**DOI:** 10.1021/acs.jcim.4c01203

**Published:** 2024-10-22

**Authors:** João Gabriel Gralha, André Silva Pimentel

**Affiliations:** Departamento de Química, Pontifícia Universidade Católica do Rio de Janeiro, Rio de Janeiro, RJ 22453-900, Brazil

## Abstract

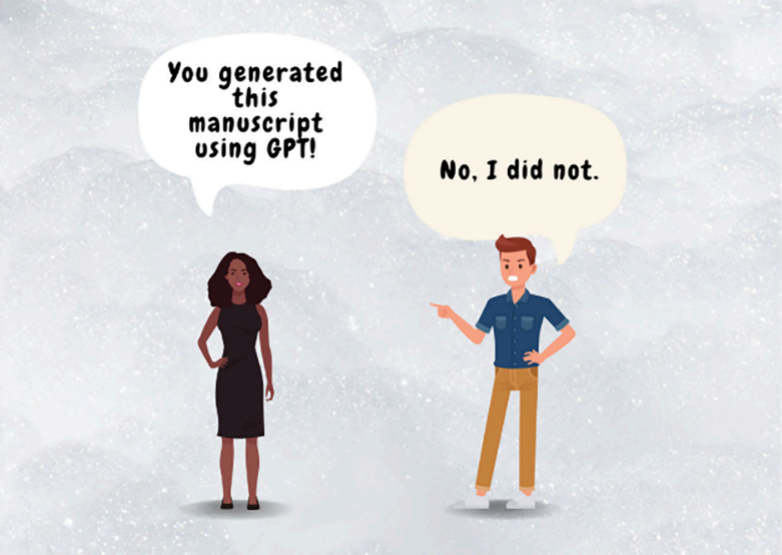

This application note explores how to address a challenging problem faced by many academics and publishing professionals in recent years: ensuring the integrity of academic writing in universities and publishing houses due to advances in Artificial Intelligence (AI). It distinguishes AI- and human-generated English manuscripts using classifier models such as decision tree, random forest, extra trees, and AdaBoost. It utilizes Scikit learn libraries to provide statistics (precision, accuracy, recall, F1, MCC, and Cohen’s kappa scores) and the confusion matrix to guarantee confidence to the user. The accuracy of the model evaluation for classification ranges from 0.97 to 0.99. There is a text data set of approximately 400 AI-generated texts and around 400 human-generated texts used for training and testing (50/50 random split). The AI texts were generated using detailed prompts that describe the text format of abstracts, introductions, discussions, and conclusions of scientific manuscripts in specific subjects. The tutorials for Gotcha GPT are written in Python by using the highly versatile Google Colaboratory platform. They are made freely available via GitHub (https://github.com/andresilvapimentel/Gotcha-GPT).

## Introduction

Who has not faced the discomfort of doubting the authenticity of a text written by a student, believing that the text could be a simple copy from the literature or the Internet without proper citation? This feeling has become an old-fashioned experience with advances in AI. Nowadays, students prompt questions and even request AI to generate all the texts needed to complete their homework quickly and easily.^1−3^ In another situation, researchers are usually asked to review a scientific manuscript and wonder when reading the text:^4−6^ Wait a minute! This is a generic text, without bibliographical references, decontextualized, quite shallow that flows well, with smooth transitions between sentences, with word repetitions, and unusual words.^2,7^

In all situations, the discomfort is obvious because the professional needs to accuse illegal conduct due to a lack of ethics, integrity, and originality of the academic manuscript.^8^ The implications of this act are evident as it may lead to legal action, and there is a need for evidence against illegal conduct. It is therefore crucial to have reliable, precise, and accurate tools for detecting texts generated by AI.^9−12^ The tools exist both for free and in paid form.^12^ However, from the point of view of the authors and the scientific community, the current tools to date may not be precise and accurate and may not agree with each other for detecting AI-generated texts. Obviously, this situation can improve with the advancement of AI and the training of tools using the texts that users upload to the platforms to verify the generation of text by AI. It is not our intention to perform a comprehensive comparison here because the web tools are intended to detect long AI-generated texts (the larger the text, the better the performance). However, our tool is tailored to detect shorter AI-generated texts than 1024 tokens (about 700 characters). Consequently, the reader must be careful, as any comparison of both approaches may lead incorrect conclusions.

Here, some issues arise with loading manuscripts into text detection platforms generated by AI. When a reviewer is invited to review a research paper, it is important for the reviewer to treat the manuscript as confidential. It is not allowed to upload the submitted manuscript or any part of it into a generative AI tool, as it could violate the confidentiality and proprietary rights of the author. Additionally, if the manuscript contains personally identifiable information, it may also breach the data privacy rights.

So, how can an educator or reviewer produce evidence of illegal conduct without relying on their own potentially erroneous judgment?^13^ It is clear that it is necessary to use a reliable tool with statistical metrics that does not rely on virtual training. The objective of this application is to provide the academic community with a simple and reliable tool to discriminate short texts of up to 1024 tokens (about 700 words) in English. This will allow educators, editors, and reviewers to rely on scientifically proven evidence when accusing individuals of misusing AI to write scientific manuscripts. Although we perform a simple comparison of tools and test the discrimination of several humanized AI-generated texts, it is not the purpose of this study to perform a comprehensive comparison of online tools with our tool and discriminate AI- and humanized AI-generated texts.

## Methodology

The methodology used in developing this tool was to acquire a relatively large quantity (approximately 800) of texts generated by AI using ChatGPT 3.5 and humans on subjects of interest to the public of the *Journal of Chemical Information and Modeling* and the *Journal of Chemical Theory and Computation*, in first place, but also covers topics related to *TheJournal of Physical Chemistry A/B/C* and *Letters*, *ACS Physical Chemistry Au*, *Chemical Research on Toxicology*, and *Journal of Medicinal Chemistry*. The AI texts were generated using detailed prompts (shown in Supporting Information) that describe the text format of abstracts, introductions, discussions, and conclusions of scientific manuscripts in specific subjects. The user must only inform the subject in the prompt. Subsequently, the texts were tokenized using the Generative Pretrained Transformer 2 (GPT-2), the smallest version of GPT-2, with 124 M parameters. After tokenizing the texts and calculating the number of tokens, characters, words, sentences, sentence length, and the average and standard deviation of sentence length, two metrics were calculated to discriminate the texts: perplexity and burstiness.

**Figure 1:**
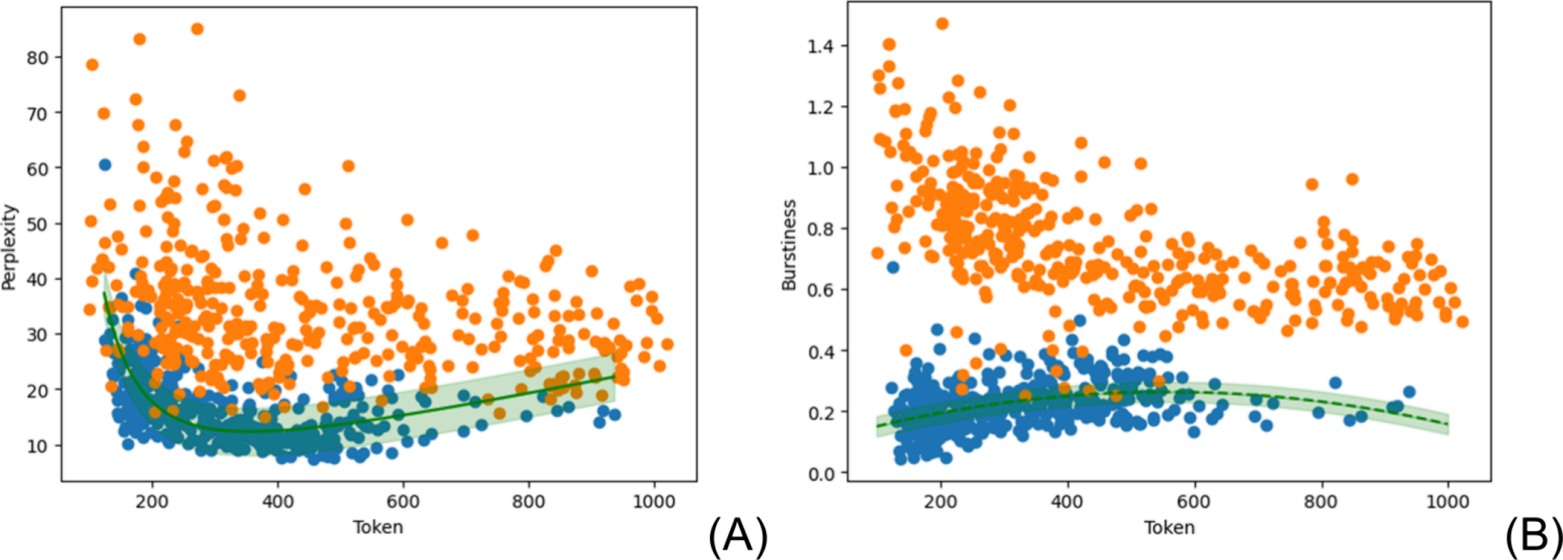
(A) Perplexity is nonlinearly related to tokens. The green line is a nonlinear best fit (y = a/x^2^ + bx, where y is perplexity and x is token). (B) Burstiness is fitted by a second-order polynomial of tokens (y′ = a′x^2^ + b′x + c′, where y′ is burstiness, and x is token). The green line is a best fit with the light green region formed by the standard deviation (±σ). Human-generated texts are represented by orange circles, and blue circles represent AI-generated texts.

Perplexity^14−16^ and burstiness^14,17,18^ are metrics used to evaluate different aspects of language models and text generation. While both involve tokens, they serve different purposes, and their correlation with tokens differs in nature and context. Perplexity measures how well a probability distribution or a language model predicts a sample. In the context of language models, it evaluates how well the model predicts the next token in a sequence of words. It is calculated as the exponentiated average negative log-likelihood of the predicted tokens:
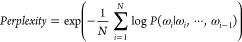
where ω*_i_* are the tokens, *N* is the number of tokens,  and is the probability of the token given the preceding tokens. Perplexity is related to tokens (in a nonlinear fashion, see Figure 1A) in the sense that it measures the probability of sequences of tokens. Lower perplexity indicates a model that better predicts the token sequence, suggesting that the model has a good understanding of the token distribution in the text.

Burstiness refers to the property of tokens that occur in clusters or bursts rather than being evenly distributed over time. In the text, burstiness can be seen as the clustering of certain words or tokens. There are different ways to measure burstiness, but one common approach is to look at the variance-to-mean ratio of token occurrences as follows:
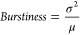
where σ^2^ is the variance, μ and is the mean occurrence rate of tokens. Burstiness is also related to tokens (in a nonlinear fashion like perplexity) by examining the distribution and frequency of tokens in a text. A higher burstiness indicates that certain tokens appear in clusters more frequently than others. There is no straightforward or direct correlation between perplexity and burstiness. Perplexity focuses on the predictive capability of a model over a sequence of tokens, while burstiness looks at the distribution pattern of tokens. There could be an indirect relationship in certain contexts. For instance, a model that handles bursty text well might show a lower perplexity for that type of text if it captures the patterns effectively. However, this is highly context-dependent and varies based on the nature of the text and the model.

In fact, perplexity and burstiness are both related to tokens but in different ways. Perplexity measures how well a model predicts tokens, while burstiness measures the clustering of token occurrences. Although there is not a direct correlation between the two tokens (Figure 1A), they may provide complementary insights into the properties of text and the performance of language models. Figure 1A shows that perplexity discriminates both human- and AI-generated texts over the tokens, but it still lacks some correlation with other variables not included in this bidimensional plot representation. So, the number of tokens, characters, words, sentences, sentence length, the average and standard deviation of sentence length, perplexity, and burstiness were calculated to classify the texts as human- and AI-generated texts using logistic regression, random forests,^19^ extra trees,^20^ decision trees,^19^ and AdaBoost classifiers.^21^ We anticipate here that all classifiers provided similar metrics (Table S1), and we decided to only present random forest classification. We also present in advance that principal component analysis^22^ was used to understand the dimensionality of the problem by calculating the cumulative variance of each parameter in the model.

We present the feature characterization of texts (the number of tokens, characters, words, sentences, sentence length, the average and standard deviation of sentence length, perplexity, and burstiness) in the Supporting Information. Figure S1 shows the distributions of important features in AI- and human-generated texts. Although tokens, perplexity, and burstiness are important to discriminate AI- and human-generated texts, mean and standard deviation of sentence length are also important, but not to the same extent to discriminate the texts (compare Figure S1(E)–(H)). The average number of words, characters, and sentences are unimportant to discriminate AI- and human-generated texts. The number of words of AI texts is 261.3, compared to 354.9 for the human-written texts, and the average number of sentences is 11.6 for the AI texts and 16.5 for the human texts. The distribution of these features is presented in Figure S1(A)–(D). It is important to mention that the average of words and the average number of sentences are not reasonable measures of the generalizability and robustness of the proposed approach. The distribution of number of words spans over a large range of tokens within the tokens allowed by the method (limit of 1024), so the distribution is more important than the average to measure the generalizability and robustness of our proposed method. The average number of sentences and its standard deviation for the AI-generated texts are fingerprints to discriminate them from human-generated texts. Usually, standard deviation of the number of sentences for the AI-generated texts is smaller than for human-generated texts, indicating a lower variation of the number of sentences in AI-generated texts. Our tool is surely recognizing texts with a lower variation of the number of sentences as AI-generated ones, rather than detecting AI-written content based on more nuanced linguistic features as explained later.

Figure 1B shows the four significant dimensions of the problem to explain the variance with >95% confidence (dashed red line). Another good practice used in the tool development was obtaining a well-balanced text data set with an approximate number of texts generated by humans and AI.^23,24^ Anyway, the resample technique may be used to ensure the balance of the data set.

The full text data set was split 50/50 to build the training and testing data sets (around 400 for human-generated texts and around 400 for AI-generated texts, excluding texts with more than 1024 tokens, which is the limit for the GPT-2 model). It is important to mention that texts with more than 1024 token index sequence lengths are longer than the specified maximum sequence length for this model, and running this sequence through the model may result in indexing errors. It is also important to emphasize that the features were scaled in the classification pipeline using the standard scaler class of the sklearn package. The text data set was balanced using the sklearn resample class.

## Usage

In the code for training and testing, the data set is uploaded by using DOCX word files that are converted to TXT files (texts files in PDF format may be converted into TXT files; however, this is not a good practice because the conversion generates some unreadable characters and new line character ‘/n’ in the end of each line in the TXT files). This generates a pandas data frame with the characteristics of the texts. Important features may be filtered using dimensionality reduction through principal component analysis; however, the number of tokens, perplexity, and burstiness are the most important parameters to discriminate the AI- and human-generated texts. Afterward, a classification model for the texts present in the data set is created by training. Then the model is tested using the characteristics of other new texts to produce statistical metrics and a confusion matrix. The model is saved and downloaded for use in the detection code for the texts supplied by the user.

In the detection code, the model is uploaded in the root of the Google Colab notebook. The texts supplied by user in DOCX format are converted into TXT files, and a data frame is built with the characteristics of the texts. Dimensionality reduction with principal component analysis may be used if necessary. Then, the model utilizes the important characteristics to predict whether the text is AI-generated or human generated.

## Results and Discussions

**Figure 2:**
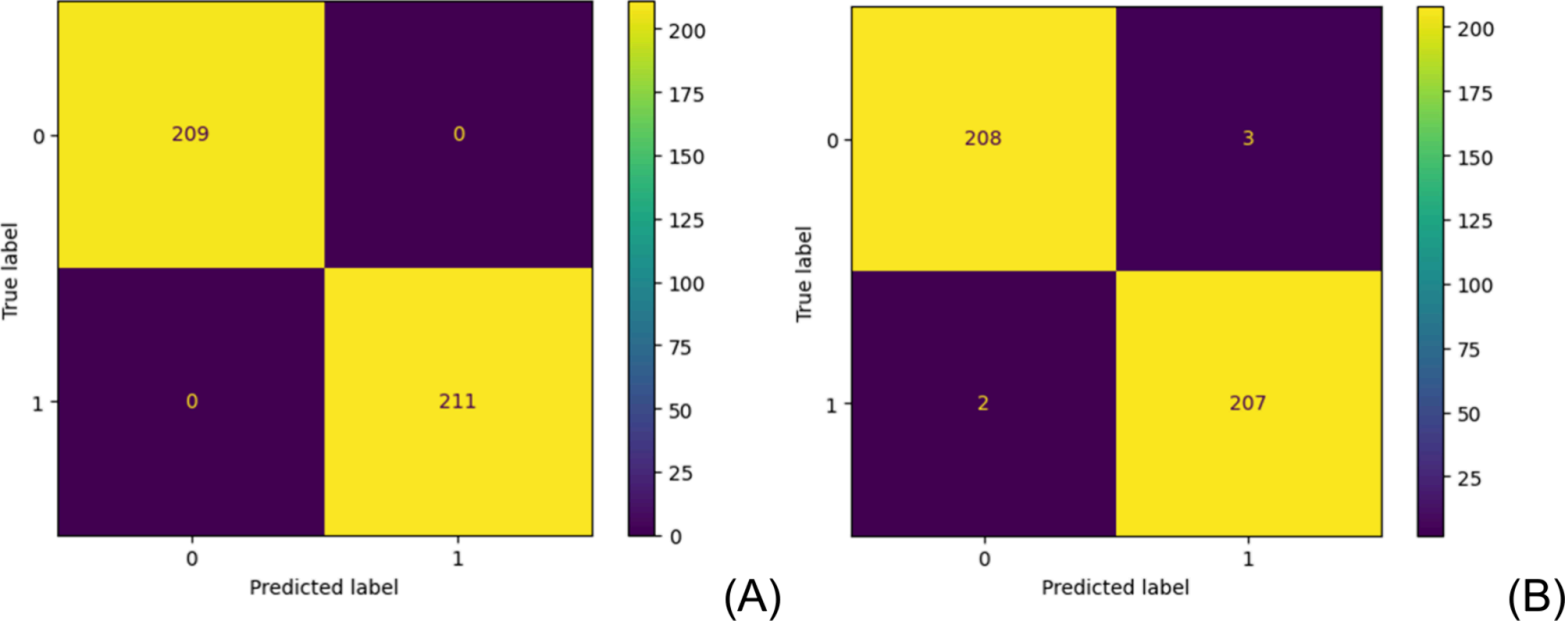
Confusion matrices for (A) training and testing (B) data sets to classify the human- and AI-generated texts (“0” means human and “1” is AI) using the random forest classifier after resampling the data set. The metrics^25,26^ of precision, recall, F1 score, accuracy, Matthew correlation coefficient (MCC), and Cohen’s kappa (κ) for training classification are all 1.00 (except AdaBoost, see Table S1) and for the testing classification are precision: 0.986; recall: 0.990; F1 score: 0.988; accuracy: 0.988; MCC: 0.976; and κ: 0.976.

All classifiers used in this tool show similar results, with random forest, extra trees, and decision tree being slightly superior in precision scores (see Table S1). The classifier models^19^ were built and saved to provide users with an easier and faster way to classify their own texts without the need for training. Here, we show only the results for the random forest model. Figure 2 presents the confusion matrices for (A) training and testing (B) data sets to classify the human- and AI-generated texts (“0” means human and “1” is AI) using the random forest classifier. Figure S2 also shows the confusion matrices using the extra tree, decision tree, and AdaABoost classifiers to confirm that all classifier models give similar results in terms of metrics (Table S1). Table S1 presents the precision, recall, F1 score, accuracy, Matthew correlation coefficient (MCC), and Cohen’s kappa coefficient (κ) for testing the classification models. For the training data set, it is observed that there are neither false positives or false negatives, indicating a perfect classification model. Figure 2B shows that there are only 2 false positives out of 209 AI-generated texts (0.9%) and 3 false negatives out of 211 human-generated texts (1.4%). These classifications were found to have a precision of 0.986 and an accuracy of 0.988. The Matthew correlation coefficient (MCC) and Cohen’s kappa κ are specific metrics for robust classification models. Both were found to be very high, 0.976, indicating an excellent classification model. It is possible to conclude that our classification model was successful in classifying human- and AI-generated texts. We performed the classification without the number of tokens to understand whether the model depends on this parameter. We found that the model performs very well with an accuracy and precision higher than 0.995. When all of the features are included in the model training, the precision and accuracy in the testing are slightly lower, 0.986. Also, we used around 800 AI- and human-generated texts with about the same number of tokens (around 300 ± 100 tokens) to verify if the model performs well discriminating texts of similar size. We also found that the model discriminates very well with accuracy and precision greater than 0.97 in this case.

We present a comparison of some online detectors of AI-generated texts (GPTZero,^27^ ZeroGPT,^28^ Writer AI,^29^ and Hive AI^30^) with Gotcha GPT using AI- and human-generated texts in the Supporting Information (Table S2). Although we emphasize that online detectors are developed to detect longer texts than Gotcha GPT is tailored to detect, our tool performed much better than the detectors ZeroGPT and Writer AI for evaluated texts (shorter than 1024 tokens). GPTZero and Hive AI are as efficient as Gotcha GPT for detecting AI-generated texts. However, ZeroGPT, Writer AI, Hive Ai, and Gotcha GPT performed better than did GPTZero for detecting human-generated texts.

In the opinion of the authors, it is possible to draw some insights after analyzing the classification of human- and AI-generated texts. It is necessary to have a large amount of data (around 1000 texts in a well-balanced data set) in order to statistically discriminate between the two types of texts. The classification model is trained and tested using English-written texts, so the trained model in English should not work for detecting different languages.^31^ However, we trained a model for discriminating between AI- and human-generated Portuguese texts that works just as well as it does for English texts, even though Portuguese texts seem to be more complex and difficult to discriminate using the same approach. The precision, recall, F1, accuracy, MCC, and κ scores for the testing classification of Portuguese texts are slightly lower than those for English texts. Therefore, if properly trained in different languages, then it can be used for the task. Although it is also trained and tested with specific subjects chosen by the authors, the authors believe that it should work for different subjects as shown in preliminary tests. However, it is very simple to upload 1 to 4 paragraphs of manuscripts (limited to 1024 tokens) to train a model for different subjects. We also trained the model using ChatGPT 3.5, but we did not test it with different versions of ChatGPT or even different chatbots. It is important to note that the advancements in AI pose another potential future challenge and treat: the development of tools to humanize AI-generated texts, creating texts very similar to the ones generated by humans. The issue of humanized AI texts^32^ requires a different approach than the classification method presented here. It is important to emphasize that our classification model is developed to discriminate between human- and AI-generated texts and not between human- and humanized AI-generated texts. Also, our tool is not tailored to classify AI-generated texts that are further modified by humans to deceive readers. As the existing tools for humanizing AI-generated texts are still being developed and are not effective up to date of first submission of this manuscript, this issue was not considered in the first draft of this application note, although we tried with no success. However, this field has been developed while our manuscript was being refereed. The free online HumanizeAi tool^33^ exceled in rephrasing AI-generated texts, eliminating any robotic undertones. Furthermore, the developer of the Humanize AI tool guarantees the humanized text is 100% original and bypasses all AI detection systems currently available. As the issue of humanized text is essential for the topic being addressed, it is an important test to indicate whether the Gotcha GPT is robust to detect humanized AI-generated texts. In addition, journals or funding agencies likely receive submissions that were generated by AI systems but which were edited by humans or generated after multiple interactions with GPT tools such as ChatGPT. Therefore, we also tested whether Gotcha GPT can still recognize an AI-generated text after it was AI- or human edited once. Using the DOCX files with AI- and humanized AI-texts, we found that Gotcha GPT detects 100% of the AI-generated texts and 90% of the humanized AI-generated text after the humanization process is performed once as presented in Table S3 (the hit rate for detection using PDF texts was 32/40 = 80%, indicating it is better to use DOCX files because PDF to text conversion yields nonrecognizable characters and new line character ‘/n’). The developer of this humanization tool suggests that the user may get an AI-generated text indistinguishable from human writing after performing the task a couple of times.

Our tool may be recognizing texts with longer sentences (Figure S1(E)) as AI-generated content rather than detecting AI-written content based on more nuanced linguistic features. We tried to detect AI-generated content based on more nuanced linguistic features (detection of linking words such as although, because, since, unless, while, whereas, therefore, however, moreover, furthermore, consequently, nevertheless, thus, hence, accordingly, meanwhile, similarly, likewise, otherwise, instead, nonetheless, additionally). Although the linking words may be features that can be used to discriminate AI- and human-generated texts, as presented in Figure S3, the classifier was not able to discriminate human- and AI-generate texts with greater accuracy (see Figure S4) compared with other features (the number of tokens, perplexity, and burstiness) presented in our study.

Finally, the advantage of our tool is explained as follows. There is no need to upload the files on the Internet, ensuring the confidentiality and proprietary rights of the authors and adhering to the publishing ethics of the journal in the peer review process.

## Limitations and Cautions

Unreliable detectors may cause embarrassing situations, especially when there is a high probability of false positives. Professors should use as many AI detectors as possible in higher education and seek judgment from a second AI specialist to avoid false cheating accusations. Accusing authors of using AI tools when they have written the content themselves may damage valuable relationships, such as professor–student relationships, and may lead to legal actions between academics and publishing houses (or universities). In both cases, it is possible to ruin the career of a young researcher by raising an incorrect flag. That is why it is crucial not to rely solely on AI detectors.

To date, it is difficult to find a tool that reliably identifies both AI- and human-generated texts. AI detection is still in its early stages, and currently AI detection tools are behind text generative pretrained transformers. However, they are no replacement for human judgment, especially when young researchers are being observed for illegal conduct. In the opinion of the authors, due to the reliability of AI detectors, it is a good practice to flag the researcher in the first instance of illegal conduct, warn the researcher in the second instance, and then act rigorously in the third instance. In the case of higher education, a lack of trust in students creates discomfort between professors and students, which has a negative impact on the classroom.

Our AI detector seems to perform well with texts from scientific journals on which it was not trained on. However, it seems to be highly specialized for scientific journal articles written in English. Indeed, it has excellent performance for paragraphs, but it may fail for different structures, such as a list of markers, which is very common in AI-generated texts. When presented with texts with different structures such as nonscientific manuscripts, it may fail to recognize them as being written by humans or AI, and may be confounded, because it is not trained to perform this task. Finally, it was not trained to discriminate between AI- and human-generated texts from languages other than English. However, it can be easily trained using the same code, but the user must create the data set with a user-defined language. Preliminarily, we trained a model with Portuguese manuscripts, which have a structure that is more complex than that of English texts. The results were very satisfactory (precision: 0.967; recall: 1.000; F1 score: 0.984; accuracy: 0.984; MCC: 0.968; and κ: 0.968; see Table S4 and Figure S5), but it is important to train and test with different data sets to achieve better reliability.

## Conclusion

The Gotcha GPT application was developed to help academics detect AI- and human-generated manuscripts. The metrics used to evaluate the detection resulted in scores of 0.97–0.99, which is excellent and provides reliability for the application. However, the use of AI-based paraphrasing tools may make AI content nearly undetectable in many cases, and human writing can also be incorrectly flagged as AI-generated text because humans may write like machine sometimes. It is important to use human judgment to avoid accusations of illegal conduct and to use different signals of AI to contend such as repetition of words, repetitive sentence structure, unnatural word usage, tortured acronyms, generic or impersonal tone, contradictory statements, inconsistent verb tense, and stiff, formal, or matter-of-fact writing style. Combining AI detectors and the signaling of AI content could help uncover misconduct in academic manuscripts.

## Data Availability

Code, model, and processing scripts for this paper are available at github (https://github.com/andresilvapimentel/gotcha-gpt). The data analysis scripts and tutorials of this application are also available in the interactive notebook in Google Colaboratory platform.

## Supporting Information Available

Prompts to generate texts of abstracts, introductions, discussions, and conclusions. Feature distributions for AI- and human-generated texts. Confusion matrices for training and testing data sets to classify the human- and AI-generated English and Portuguese texts using the extra trees, AdaBoost, and decision tree classifiers. Metrics of precision, recall, F1 score, accuracy, Matthew correlation coefficient, and Cohen’s kappa for training and testing data sets to classify the human- and AI-generated English and Portuguese texts using the Random Forest, Extra Trees, AdaBoost, and Decision Tree classifiers after resampling the data set. Comparison of some online detectors of AI-generated texts with Gotcha GPT using AI- and human-generated English texts. Robustness test of Gotcha GPT against humanized AI-generated English texts. Distribution of linking words in AI- and human-generated English texts. Confusion matrices for training and testing data sets to classify the human- and AI-generated English texts using the Random Forest classifier of the model including linking words. (PDF)
