# Supplementary material for: Gotcha GPT: Ensuring the Integrity in Academic Writing
Source: J Chem Inf Model. 2024 Oct 22;64(21):8091–7. doi: 10.1021/acs.jcim.4c01203 (PMC11558662; doi:10.1021/acs.jcim.4c01203)
Supplement: Supplementary file 1 — ci4c01203_si_001.pdf [file ci4c01203_si_001.pdf]

Gotcha GPT: Ensuring the Integrity in Academic Writing

João Gabriel Gralha and André Silva Pimentel*

Departamento de Química, Pontifícia Universidade Católica do Rio de Janeiro, Rio de Janeiro, RJ 22453-900 Brazil

* E-mail: a_pimentel@puc-rio.br

Supplementary Material

The AI texts were generated using detailed prompts (shown below) that describes the text format of abstracts, introductions, discussions, and conclusions of scientific manuscripts in specific subjects. The user must only inform the subject in the prompt. The subjects were chosen on subjects of interest to the public of the Journal of Chemical Information and Modeling and the Journal of Chemical Theory and Computation, in first place, but also covers topics related to the Journal of Physical Chemistry A/B/C and Letters, ACS Physical Chemistry Au, Chemical Research on Toxicology, and Journal of Medicinal Chemistry.

Prompt to generate abstracts

Prepare a short abstract in one paragraph format about the study "*subject*" providing a concise summary of the study, allowing readers to quickly understand the purpose, methods, results, and conclusions. Describe the main purpose or aim of the study. Clearly state what problem or question the study addresses. Explain the methodology used in the study. Briefly mention the research design, data collection methods, and any statistical techniques employed. Provide a concise summary of findings. Highlight key results, significant trends, and important conclusions. Conclude the abstract by briefly discussing the implications of your results in real life, industry and society. Explain what the findings mean in the context of the field and explain any limitations or areas for further research.

Prompt to generate introductions

Prepare a brief introduction of about 50 lines about the topic "*subject*". This introduction must contain the following aspects: the background and recent advances and applications to new chemical problems or new molecules; the modeling study leading to a design that enables new and successful experiments irrespective of the modeling technique; models of an important new endpoints and demonstrably better models (e.g., larger and/or much higher quality) than any other existing models; must be a provocative introduction criticizing/rejecting existing paradigms; very well grounded and preferably must provide the reader some idea(s) how to solve the problem; and the introduction must outline the perspectives, motivations, and main objectives of the study. The introduction must describe new approaches for modeling, clustering, and similarity estimation, must be benchmarked against multiple different tasks and consistently show superiority. These approaches could be developed and tested using simulated data sets but applied to several real tasks, and

actual compounds should be designed using them. The introduction must describe data sets, should contain some unique features such as the biggest existing data set and user-friendly database.

Prompt to generate discussions

Prepare a brief discussion of about one page (two or three paragraphs) about the limitations, advantages and disadvantages on the topic "*subject*". This discussion must contain the following aspects: must be a provocative discussion criticizing/rejecting existing paradigms; very well grounded and preferably must provide the reader some details on the limitations, advantages and disadvantages.

Prompt to generate conclusions

Prepare a short conclusion about the study "*subject*" in one paragraph format to leave a lasting impression for the reader. Begin by reminding the reader of the research problem and the main focus of study. Avoid using words like "In conclusion" or "To conclude." Instead, find a novel way to circle back to the problem from the more detailed ideas of the study. Summarize the study addressing the problem. Summarize the overall findings. Discuss the implications of the study explaining why the study matter and what impact it has. Connect the findings to broader contexts or real-world applications.

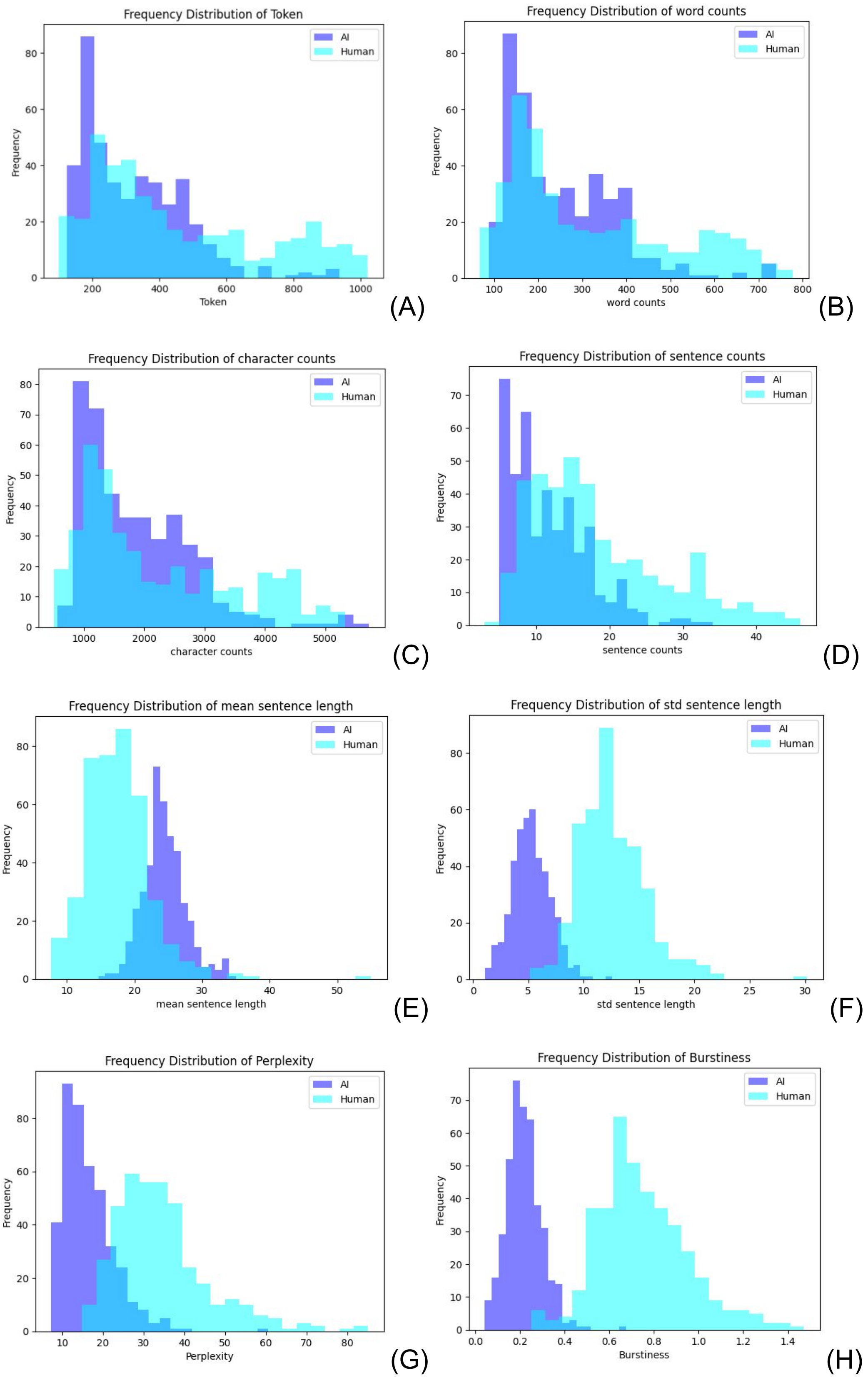

Figure S1. The feature distributions for AI- and human-generated texts. (A) token; (B) word counts; (C) character counts; (D) sentence counts; (E) mean sentence length; (F) standard deviation of sentence length; (G) perplexity; and (H) burstiness.

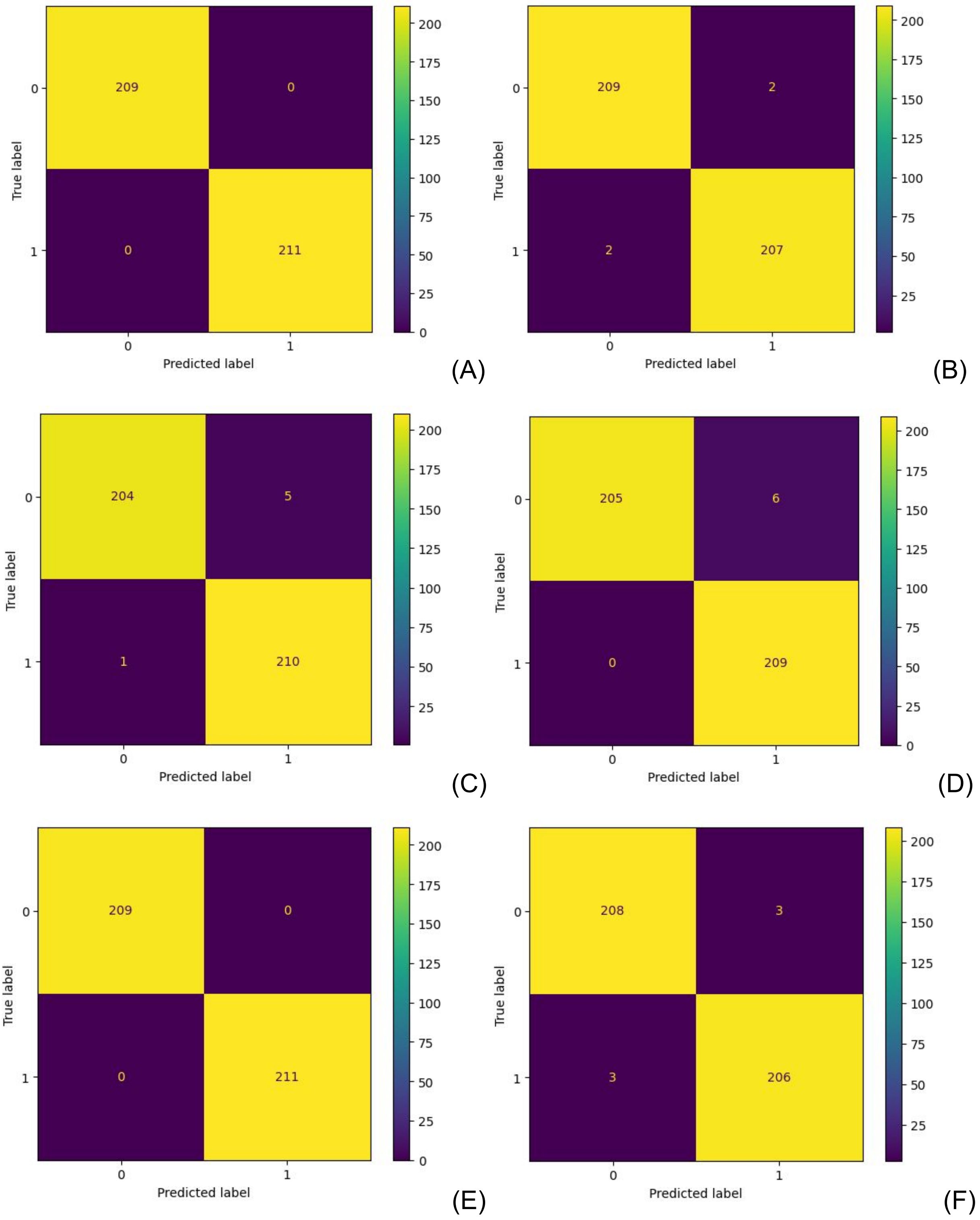

Figure S2. The confusion matrices for training (left column) and testing (right column) datasets to classify the human- and AI-generated English texts ("0" means human and "1" is AI) using the Extra Trees (A and B), AdaBoost (C and D), and Decision Tree (E and F) classifiers.

Table S1. The metrics of precision, recall, F1 score, accuracy, Matthew correlation coefficient (MCC), and Cohen´kappa (κ) for training and testing datasets to classify the human- and AI-generated English texts (“0” means human and “1” is AI) using the Random Forest (RF), Extra Trees (ET), AdaBoost (AB), and Decision Tree (DT) classifiers after resampling the dataset.

Dataset	Models	Scores					
		Precision	Recall	F1	Accuracy	MCC	κ
Training	RF	1.000	1.000	1.000	1.000	1.000	1.000
	ET	1.000	1.000	1.000	1.000	1.000	1.000
	AB	0.977	0.995	0.986	0.986	0.972	0.971
	DT	1.000	1.000	1.000	1.000	1.000	1.000
Testing	RF	0.986	0.990	0.988	0.988	0.976	0.976
	ET	0.990	0.990	0.990	0.990	0.981	0.981
	AB	0.972	1.000	0.986	0.986	0.972	0.971
	DT	0.986	0.986	0.986	0.986	0.971	0.971

Table S2. Comparison of some online detectors of AI-generated texts with Gotcha GPT using AI- and human-generated English texts.

Text	GPTZero	ZeroGPT	Writer AI	Hive AI	GotchaGPT
abstract_ai_1	100% AI	0% AI	83% Human	29.2% AI	100% AI
abstract_ai_2	100% AI	36% AI	79% Human	99.9% AI	100% AI
abstract_ai_3	100% AI	0% AI	69% Human	99.9% AI	100% AI
abstract_ai_4	100% AI	0% AI	84% Human	99.9% AI	100% AI
abstract_ai_5	100% AI	0% AI	79% Human	99.9% AI	100% AI
conclusion_ai_1	100% AI	8% AI	87% Human	97.7% AI	100% AI
conclusion_ai_2	100% AI	16% AI	86% Human	99.9% AI	100% AI
conclusion_ai_3	100% AI	9% AI	74% Human	99.9% AI	100% AI
conclusion_ai_4	100% AI	0% AI	87% Human	99.9% AI	100% AI
conclusion_ai_5	100% AI	0% AI	82% Human	99.9% AI	100% AI
discussion_ai_1	100% AI	13% AI	73% Human	99.9% AI	100% AI
discussion_ai_2	100% AI	19% AI	71% Human	99.9% AI	100% AI
discussion_ai_3	100% AI	29% AI	75% Human	99.9% AI	100% AI
discussion_ai_4	100% AI	15% AI	68% Human	99.9% AI	100% AI
discussion_ai_5	100% AI	2% AI	77% Human	99.9% AI	100% AI
introduction_ai_1	98% AI	23% AI	79% Human	99.9% AI	100% AI
introduction_ai_2	93% AI	55% AI	77% Human	99.9% AI	100% AI
introduction_ai_3	100% AI	30% AI	68% Human	99.9% AI	100% AI
introduction_ai_4	100% AI	7% AI	72% Human	99.9% AI	100% AI
introduction_ai_5	100% AI	6% AI	72% Human	99.9% AI	100% AI
abstract_human_1	92% Human	100% Human	100% Human	100% Human	100% Human
abstract_human_2	97% Human	100% Human	95% Human	100% Human	100% Human
abstract_human_3	99% Human	100% Human	100% Human	100% Human	100% Human
abstract_human_4	99% Human	100% Human	100% Human	100% Human	100% Human
abstract_human_5	97% Human	100% Human	100% Human	100% Human	100% Human
abstract_human_6	99% Human	100% Human	100% Human	100% Human	100% Human
abstract_human_7	97% Human	100% Human	98% Human	100% Human	100% Human
conclusion_human_1	51% Human	100% Human	95% Human	100% Human	100% Human
conclusion_human_3	97% Human	100% Human	100% Human	100% Human	100% Human
conclusion_human_4	59% Human	100% Human	100% Human	100% Human	100% Human
conclusion_human_5	94% Human	100% Human	99% Human	100% Human	100% Human
conclusion_human_6	94% Human	100% Human	100% Human	100% Human	100% Human
conclusion_human_8	97% Human	100% Human	92% Human	100% Human	100% Human
conclusion_human_9	66% Human	100% Human	100% Human	100% Human	100% Human
introduction_human_1	88% Human	100% Human	100% Human	100% Human	100% Human
introduction_human_2	97% Human	100% Human	95% Human	100% Human	100% Human
introduction_human_3	78% Human	100% Human	100% Human	100% Human	100% Human
introduction_human_4	95% Human	99% Human	99% Human	100% Human	100% Human
introduction_human_5	89% Human	100% Human	100% Human	100% Human	100% Human

Table S3. Robustness test of Gotcha GPT against humanized AI-generated English texts.

AI-generated text	AI	Detection of humanized AI-text*	Detection of humanized AI-text**
introduction_ai_1	100%	100% AI	100% AI
introduction_ai_2	100%	100% AI	100% AI
introduction_ai_3	100%	100% AI	100% AI
introduction_ai_4	100%	100% AI	100% AI
introduction_ai_5	100%	100% AI	100% AI
introduction_ai_6	100%	100% AI	100% AI
introduction_ai_7	100%	100% AI	100% AI
introduction_ai_8	100%	100% AI	100% AI
introduction_ai_9	100%	100% AI	100% AI
introduction_ai_10	100%	100% AI	100% AI
introduction_ai_humanized_1	100%	100% AI	100% AI
introduction_ai_humanized_2	100%	100% AI	100% AI
introduction_ai_humanized_3	100%	100% AI	100% Human
introduction_ai_humanized_4	100%	100% AI	100% AI
introduction_ai_humanized_5	100%	100% AI	100% AI
introduction_ai_humanized_6	100%	100% AI	100% AI
introduction_ai_humanized_7	100%	100% AI	100% AI
introduction_ai_humanized_8	100%	100% AI	100% Human
introduction_ai_humanized_9	100%	100% AI	100% Human
introduction_ai_humanized_10	100%	100% Human	100% Human
discussion_ai_1	100%	100% AI	100% AI
discussion_ai_2	100%	100% AI	100% AI
discussion_ai_3	100%	100% AI	100% AI
discussion_ai_4	100%	100% Ai	100% AI
discussion_ai_5	100%	100% AI	100% AI
discussion_ai_6	100%	100% AI	100% AI
discussion_ai_7	100%	100% AI	100% AI
discussion_ai_8	100%	100% AI	100% AI
discussion_ai_9	100%	100% AI	100% AI
discussion_ai_10	100%	100% AI	100% AI
discussion_ai_humanized_1	100%	100% AI	100% Human
discussion_ai_humanized_2	100%	100% AI	100% Human
discussion_ai_humanized_3	100%	100% Human	100% Human
discussion_ai_humanized_4	100%	100% AI	100% AI
discussion_ai_humanized_5	100%	100% AI	100% AI
discussion_ai_humanized_6	100%	100% AI	100% AI
discussion_ai_humanized_7	100%	100% AI	100% AI
discussion_ai_humanized_8	100%	100% AI	100% Human
discussion_ai_humanized_9	100%	100% AI	100% AI
discussion_ai_humanized_10	100%	100% AI	100% AI

* using the DOCX files to convert to text files. ** using the PDF files to convert to text files.

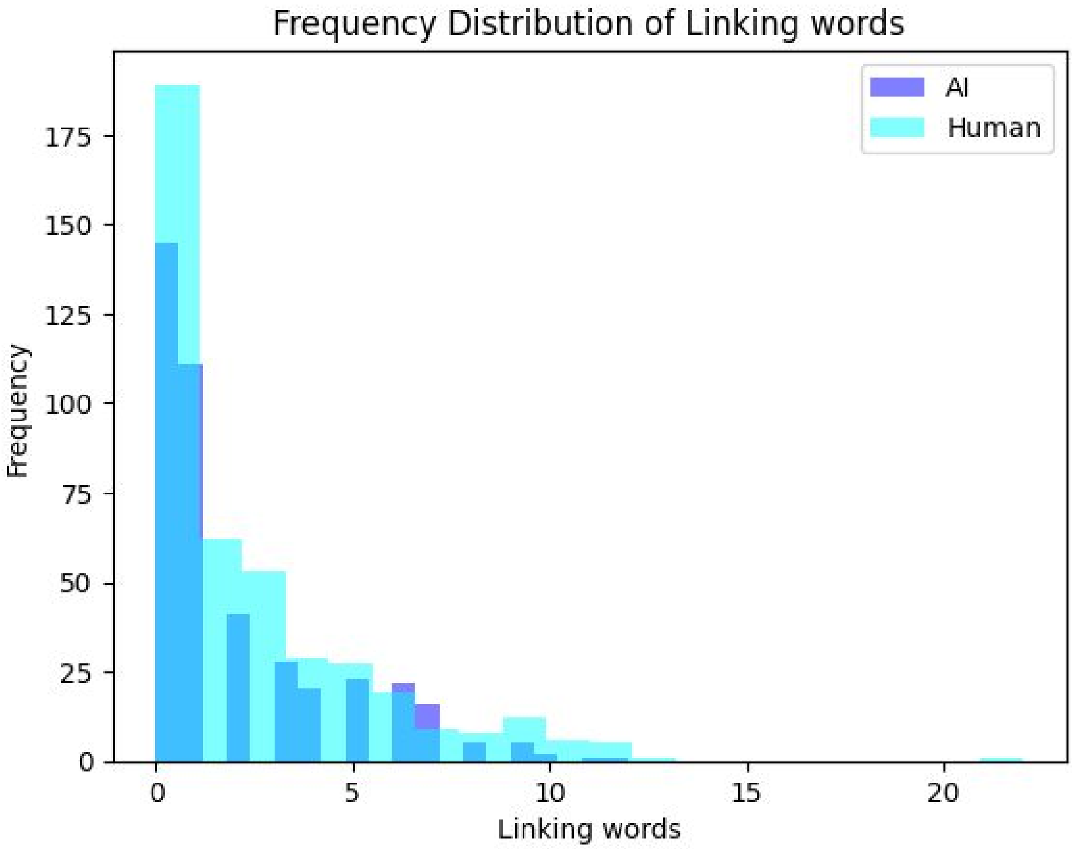

Figure S3. The distribution of linking words in AI- and human-generated English texts.

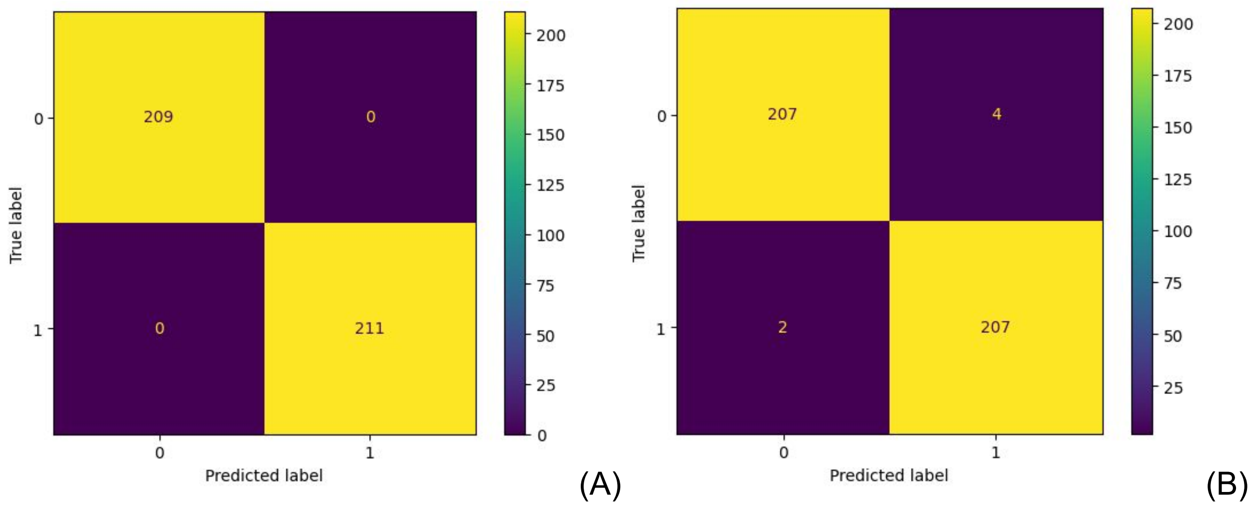

(A) (B)

Figure S4. The confusion matrices for (A) training and testing (B) datasets to classify the human- and AI-generated English texts ("0" means human and "1" is AI) using the Random Forest classifier of the model including linking words. The metrics[25,26] of precision, recall, F1 score, accuracy, Matthew correlation coefficient (MCC), and Cohen´kappa (κ) for training classification are precision: 1.000; recall: 1.000; F1 score: 1.000; accuracy: 1.000; MCC: 1.000; and κ: 1.000., and for the testing classification are precision: 0.981; recall: 0.990; F1 score: 0.986; accuracy: 0.986; MCC: 0.971; and κ: 0.971.

Table S4. The metrics of precision, recall, F1 score, accuracy, Matthew correlation coefficient (MCC), and Cohen´kappa (κ) for training and testing datasets to classify the human- and AI-generated Portuguese texts ("0" means human and "1" is AI) using the Random Rorest (RF), Extra Trees (ET), AdaBoost (AB), and Decision Tree (DT) classifiers after resampling the dataset.

Dataset	Models	Scores					
		Precision	Recall	F1	Accuracy	MCC	κ
Training	RF	1.000	1.000	1.000	1.000	1.000	1.000
	ET	1.000	1.000	1.000	1.000	1.000	1.000
	AB	1.000	1.000	1.000	1.000	1.000	1.000
	DT	1.000	1.000	1.000	1.000	1.000	1.000
Testing	RF	0.967	1.000	0.984	0.984	0.968	0.968
	ET	0.967	1.000	0.984	0.984	0.968	0.968
	AB	0.995	1.000	0.998	0.998	0.995	0.995
	DT	0.972	1.000	0.986	0.986	0.973	0.973

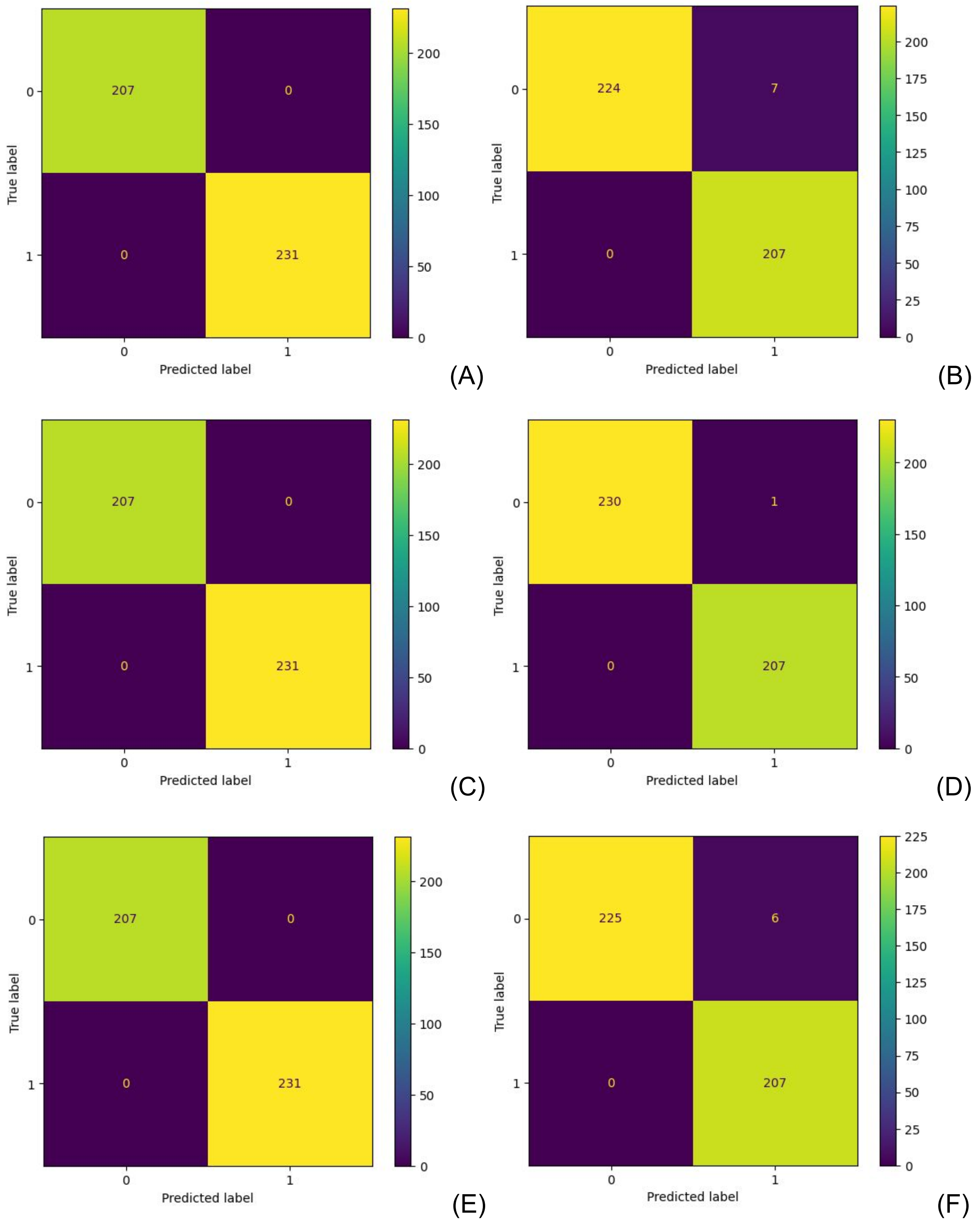

Figure S5. The confusion matrices for training (left column) and testing (right column) datasets to classify the human- and AI-generated Portuguese texts (“0” means human and “1” is AI) using the Extra Trees (A and B), AdaBoost (C and D), and Decision Tree (E and F) classifiers.
